# Mechanistic insights into ammonium-driven metabolic regulation for enhanced nemadectin biosynthesis in *Streptomyces cyaneogriseus*

**DOI:** 10.1186/s40643-026-01015-6

**Published:** 2026-01-30

**Authors:** Zishu Zhang, Junxiong Yu, Xiaoqing Song, Qingfeng Gu, Yun Zhang, Jiayun Xue, Ali Moshin, Yonghong Wang, Zejian Wang

**Affiliations:** 1 State Key Laboratory of Bioreactor Engineering, East China University of Science and Technology, 130 Meilong Rd, Shanghai, 200237 People’s Republic of China; 2 State Key Laboratory of Microbial Metabolism, School of Life Sciences and Biotechnology, Joint International Research Laboratory of Metabolic and Developmental Sciences, Shanghai Jiao Tong University, 800 Dongchuan Rd, Shanghai, 200240 The People’s Republic of China

**Keywords:** Nemadectin, *Streptomyces cyaneogriseus*, Ammonium regulation, Multi-omics analysis

## Abstract

Nemadectin, a milbemycin-class macrocyclic lactone antibiotic produced by *Streptomyces cyaneogriseus*, is a potent broad-spectrum insecticide with excellent environmental compatibility. Its derivative moxidectin, featuring a C-23 methoxime modification, demonstrates enhanced insecticidal activity and has become a commercially successful agrochemical. This study reveals ammonium regulation effectively boosts nemadectin biosynthesis in *S. cyaneogriseus*, with mechanistic insights gained through integrated multi-omics analysis. Transcriptomic profiling showed ammonium sulfate supplementation significantly upregulates the nemadectin biosynthetic gene cluster, including polyketide synthase (PKS) genes, backbone modification genes, and pathway-specific transcription factors, while also enhancing the expression of Avenolide-like signaling molecules and global transcription factor *Afskne*. Metabolomic dynamics revealed reinforced precursor biosynthesis through coordinated metabolic reprogramming: enhanced acetyl-CoA production, reinforced Embden–Meyerhof–Parnas pathway and amino acid/acyl-CoA metabolism, coupled with reduced tricarboxylic acid cycle activity. Systematic integration of physiological phenotyping, metabolite profiling, and transcriptional regulation data comprehensively elucidated the ammonium-driven overproduction mechanism, providing critical insights for developing advanced fermentation strategies and genetic engineering approaches in industrial antibiotic production.

**Graphical abstract:**

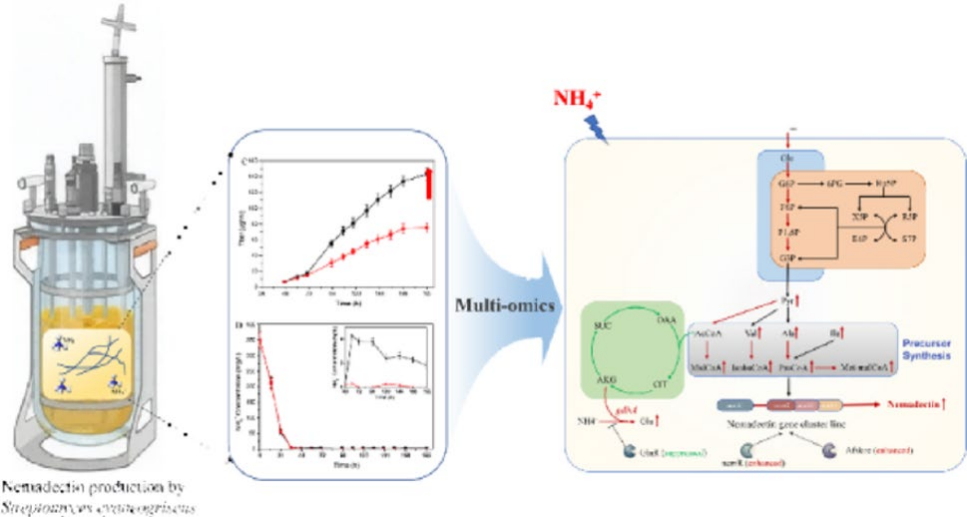

**Supplementary Information:**

The online version contains supplementary material available at 10.1186/s40643-026-01015-6.

## Introduction

Nemadectin is a potent, broad-spectrum insecticide produced by *Streptomyces cyaneogriseus*, belonging to the milbemycin subfamily of macrocyclic lactone antibiotics (Prichard et al. 2012). It exhibits excellent insecticidal effects, effectively killing various pests with decent biocompatibility and is widely used in agriculture to protect crops and livestock from infestations. The unique chemical structure of nemadectin provides a specific mechanism that reduce negative impacts on the environment. Moxidectin, a derivative of nemadectin, is chemically modified by adding a methoxime moiety at the C-23 position (Lyons et al. 1992). This chemical modification enhances the insecticidal activity of moxidectin, making it one of the widely used insecticides in the market (Doscher et al. 1989; Song et al. 2025).

Recent research has significantly advanced our understanding of nemadectin biosynthesis in *S. cyaneogriseus*. Studies elucidating its biosynthetic gene cluster and characteristics have highlighted their importance for developing novel biopesticides and optimizing production processes (Li et al. 2019; Wang et al. 2015). Whole-genome sequencing of the non-cyanogenic strain has further revealed its genetic blueprint and biosynthetic potential, providing a foundation for metabolic studies and yield improvement (Wang et al. 2015). Notably, the essential LAL-family regulator NemR was identified to directly activate key biosynthetic operons and indirectly control others through a conserved binding motif, while uncovering four novel regulatory targets; *nemR* overexpression alone increased nemadectin production by 79.9% (Li et al. 2019). Despite these genomic and regulatory insights, research on large-scale physiological metabolism and optimal fermentation process control remains limited. Further investigation is critically needed to optimize fermentation conditions, enhance production efficiency, and identify key factors governing nemadectin biosynthesis at an industrial scale.

Nitrogen sources play a crucial role in antibiotic production, as their availability and type directly influence cell growth and metabolic activity (Zhang et al. 2019). Nitrogen limitation strategies are widely applied in fermentation processes to regulate the growth rate of cells, thereby directing more energy and resources towards the synthesis of metabolic products (Chen et al. 2013). This regulation not only limits cell proliferation but also optimizes specific metabolic pathways, enhancing the synthesis of antibiotics. Studies have shown that proper management of nitrogen sources can significantly enhance both the yield and purity of antibiotics. During the early stages of antibiotic fermentation, nitrogen source consumption is primarily dedicated to supporting cell growth and reproduction. As nitrogen sources gradually decrease, the growth rate of cells also decreases. When nitrogen sources are depleted to a limiting level, the fermentation system transitions into a low-nitrogen-limiting state, triggering the cells to shift from growth to antibiotic synthesis phase (Martín et al. 2011). This stage marks a critical period, as the metabolic focus of the cells shifts from growth to product synthesis, determining the yield of antibiotics.

Ammonium salts are commonly used as nitrogen sources in antibiotic production due to their efficient microbial absorption and utilization; however, high concentrations of ammonium ions can negatively impact synthesis. For instance, excessive ammonium ions deplete biosynthetic precursors, obstructing avilamycin production (Zou et al. 2009), indicating that careful concentration control is necessary to avoid yield inhibition. Studies show ammonium ion concentration significantly influences both cell growth and antibiotic synthesis: maintaining 20–150 mM effectively promotes cell growth and gentamicin production (Technikova-Dobrova et al. 2004), while lower concentrations benefit A40926 glycopeptide antibiotic synthesis. This demonstrates that varying concentrations differentially regulate cellular metabolic pathways during fermentation, making optimization crucial for enhancing yields.

Building upon our previous discovery that ammonium regulation significantly enhances nemadectin production in *S. cyaneogriseus*, this study employs an integrated systems biology approach to elucidate the underlying molecular mechanisms. While our prior work established that online oxygen uptake rate (OUR) control, optimized via ammonium sulfate feeding during the mycelium differentiation phase (targeting ~ 17.5 mmol L^−1^h^−1^), dramatically increased titers by 129.5% (reaching 2805.1 µg mL^−1^) and scaled successfully to pilot-scale (2867.3 µg mL^−1^), the precise metabolic and genetic reprogramming driving this enhancement remained unclear (Song et al. 2025). Here, we utilize transcriptomic and metabolomic analyses to investigate the ammonium-driven changes in: (1) the nemadectin biosynthetic gene cluster expression, (2) precursor pool availability, (3) central carbon metabolic flux, and (4) ammonium assimilation pathways. This multi-omics integration comprehensively reveals the regulatory mechanism responsible for the production boost following ammonium supplementation. The insights gained provide a critical foundation for future metabolic engineering strategies to develop high-yielding strains and optimize fermentation processes for industrial-scale nemadectin production.

## Materials and methods

### Synthetic medium

Through single-factor optimization experiments and response surface methodology, a fermentation medium suitable for both microbial growth and nemadectin production was developed. The synthetic medium contained (g/L): glucose: 17.0, maltose: 7.52, ammonium sulfate: 1.59, potassium hydrogen phosphate: 0.033, calcium carbonate: 10.17, magnesium sulfate: 1.5, copper sulfate: 0.01, cobalt chloride: 0.002, zinc sulfate: 0.001, manganese sulfate: 0.001, sodium molybdate: 0.002.

### Supplementation strategy

#### Strategy for glucose solution supplementation

The sugar supplementation strategy was consistent with that used in complex media, involving periodic measurement of glucose concentration in the fermentation broth. When the glucose concentration dropped to 2 g/L, glucose solution was added, and the supplementation rate was controlled to maintain the glucose concentration around 2 g/L.

#### Strategy of ammonium sulfate solution supplementation

Ammonium sulfate solution was added when the mycelium differentiation began. By controlling the supplementation rate, it was ensured that the concentration of ammonium ions in the fermentation broth remained below 5 mg/L. The supplementation period lasted for 24 h.

### Preparation and preservation of the transcriptome-sequencing samples

After taking 5 mL of fermentation broth immediately, mycelium and medium were separated using cold centrifugation. Later supernatant was discarded and resuspended the pellet in 5 mL of sterile PBS, followed by centrifugation. This process was repeated twice. After final centrifugation the supernatant was removed and mycelium pellet was retained. Then 5 mL of RNA protection solution was added and incubated at room temperature overnight. The mixture was then stored at -80 °C. A total of 7 samples were collected, with 3 replicates for each sample.

The extraction and sequencing of total RNA for transcriptome samples were completed by BGI Genomics (Shenzhen) Co., Ltd. The quality report of the transcriptome sequencing samples is shown in Table 1. All samples passed the quality control and met the sequencing requirements of BGI Genomics. Class A indicated that the quality met the library construction and sequencing requirements, and the total amount was sufficient for two or more sequencing runs. Class B indicated that the quality met the library construction and sequencing requirements, but the total amount was sufficient for only one sequencing run and insufficient for two.

**Table 1:** Quality test results of cell samples of RNA-sequence

Serial number	Sample name	Concentration (ng/µL)	OD260/ 280	OD260/ 230	RIN	23 S/ 16 S	Result note
1	N-1-1	61	2.02	1.95	9.9	2.0	A class
2	N-1-2	54	2.05	1.84	9.5	2.1	A class
3	N-1-3	49	1.99	1.86	9.4	2.1	A class
4	N-2-1	41	1.97	1.83	9.8	2.1	B class
5	N-2-2	59	1.94	1.73	9.9	2.2	A class
6	N-2-3	41	1.78	1.29	9.9	1.9	B class
7	N-3-1	70	2.03	1.81	9.8	2.0	A class
8	N-3-2	48	2.19	1.43	7.3	1.9	B class
9	N-3-3	45	1.69	1.65	9.1	2.0	A class
10	N-4-1	27	1.92	1.82	9.3	2.0	B class
11	N-4-2	44	2.01	1.66	9.6	2.1	A class
12	N-4-3	61	2.06	1.56	10.0	2.0	A class
13	C-1-1	74	2.01	2.15	10.0	1.8	A class
14	C-1-2	79	1.94	1.54	10.0	2.0	A class
15	C-1-3	50	2.02	1.83	9.8	2.1	A class
16	N-5-1	71	2.02	1.9	10.0	2.0	A class
17	N-5-2	34	2.03	1.22	10.0	1.9	B class
18	N-5-3	72	2.05	1.79	10.0	2.0	A class
19	C-2-1	91	2.03	2.07	9.7	1.7	A class
20	C-2-2	90	2.04	1.95	9.7	1.8	A class
21	C-2-3	63	1.98	1.6	9.6	1.7	A class

### Inactivation and extraction of the metabolite group samples

Intracellular metabolite extraction was based on the reported literature, we performed rapid inactivation and extraction of intracellular major metabolites, including organic acids, amino acids, sugar phosphates, and coenzyme A derivatives (Hong et al. 2017; Liu et al. 2021). To ensure the reliability of the intracellular metabolite analysis, the IDMS (Isotope Dilution Mass Spectrometry) method was used for analysis.

Extracellular metabolite collection was completed with a rapid sampling device. We took 4 mL of fermentation broth into a 20 mL syringe containing pre-chilled steel beads. The front end of the syringe was equipped with a filter membrane. The syringe was quickly pushed to separate the mycelium from the culture liquid, after which the culture liquid was collected and stored at − 80 °C.

### Analysis of the transcriptome samples

Transcriptome sampling was conducted at five representative time points during fermentation. The first sample was taken at 36 h, when the mycelia were intact and the culture was in the vegetative growth phase (designated N-1). The second sample was collected at 41 h, when ammonium ions in the broth had just been depleted but mycelial differentiation had not yet started (designated N-2). The third sample was obtained at 48 h, marking the onset of mycelial differentiation and the transition into the nemadectin production phase (designated N-3). Immediately after this sample was taken, ammonium sulfate supplementation was initiated. The fourth sampling point was at 60 h, during which samples were collected from both the ammonium-supplemented fermenter (N-4) and the non-supplemented control fermenter (C-1). The final sampling was performed at 96 h, after ammonium supplementation had ceased and a clear difference in the specific nemadectin production rate between the two fermenters was observed; here, samples were taken from the ammonium-supplemented fermenter (N-5) and the control fermenter (C-2). For each time point, three biological replicates were collected.

To ensure comparability of gene expression levels across different genes or samples in transcriptomics analysis, the FPKM (Fragments Per Kilobase of exon model per Million mapped reads) value is typically used to measure gene expression levels (Pullan et al. 2011; Yu et al. 2022). The FPKM value represents the number of fragments mapped to each gene per million sequences, normalized to a unit of one thousand base pairs. The specific formula is as follows: 

FPKM = Number of sequencing fragments mapped to the gene’s exons/Total number of base pairs of the gene × Total number of mapped sequencing fragments

Differentially expressed genes were selected using the DEGSeq algorithm. The criteria for selecting differential genes was: (1) ∣log2RPKM ratio∣≥1.0∣ (2) *p*-value < 0.05; (3) False Discovery Rate (FDR) ≤ 0.05. Genes meeting all three criteria were considered to be differentially expressed.

## Results and discussion

### Physiological phenotype data of the fermentation process of *S. cyaneogriseus* after ammonium sulfate supplement

**Fig. 1:**
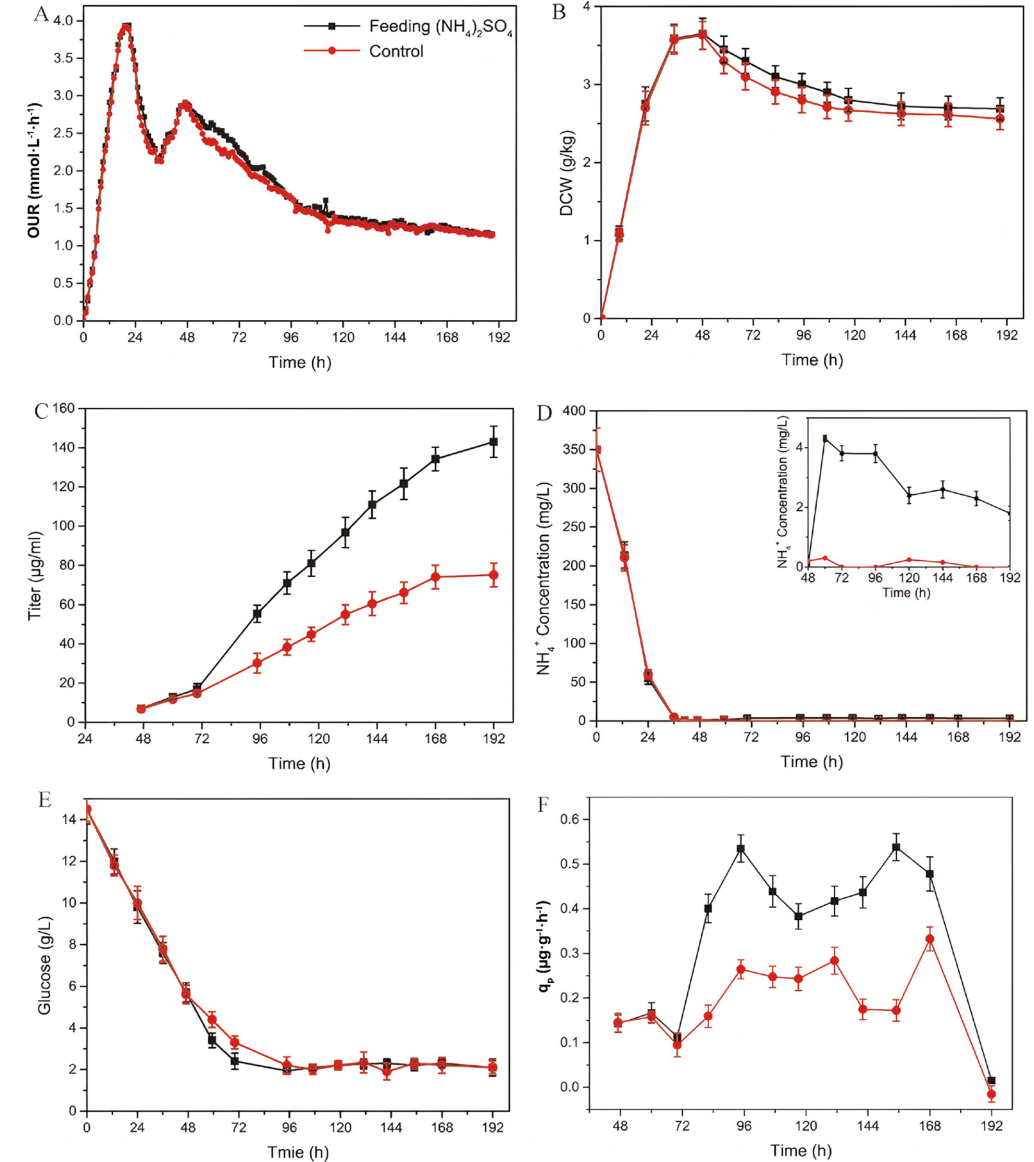
Profiles of physiological parameters for *S. cyaneogriseus* with high nemadectin production after supplementation ammonium sulfate solution. OUR (**A**), DCW (**B**), Titer (**C**), NH_4_^+^ (**D**), glucose (**E**), *q*_p_ (**F**). Data are presented as the mean ± standard deviation of three independent replicates (*n* = 3)

After the supplement of ammonium sulfate, a slight improvement in the respiratory metabolism was appeared in *S. cyaneogriseus*, and the OUR value increased to a certain extent and then restored to the control level (Fig. 1A). The dry cell weight also showed a slight increase after ammonium sulfate supplementation, accompanied by a rapid increase in glucose consumption (Fig. 1B, E). The ammonium concentration maintained at a relative higher level (3 mg/L), even though still under the ammonium ion limitation, the titer of nemadectin significantly increased following ammonium sulfate supplementation, reaching 143.1 mg/L at the end of fermentation, which increased 90.8% over the control (Fig. 1C). The specific synthesis rate of nemadectin (Fig. 1F) also showed a marked increase during production after ammonium sulfate supplementation, which was 1.2 times higher compared to that of control.

### Transcriptome analysis of *S. cyaneogriseus* after ammonium sulfate supplementation

#### Principal component analysis of gene expression and cluster analysis of gene expression patterns

**Fig. 2:**
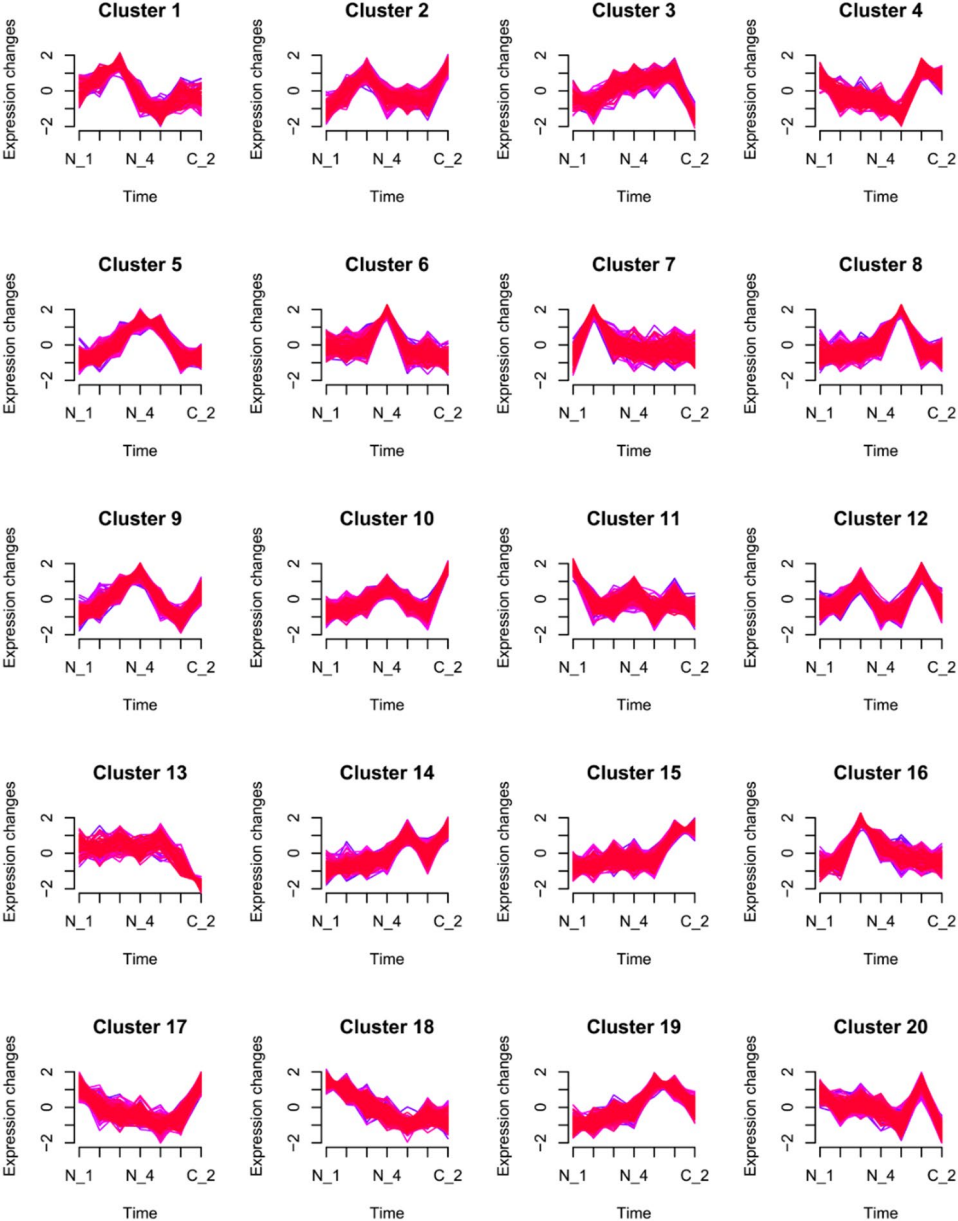
Clusters of differentially expressed genes in different culture condition. The horizontal axis is N-1, N-2, N-3, N-4, N-5, C-1 and C-2, repectively. N-1 (36 h): vegetative growth with sufficient ammonium. N-2 (41 h): ammonium depleted before differentiation. N-3 (48 h): differentiation onset, then ammonium supplementation. C‑1 & N‑4 (60 h): control and supplemented samples during supplementation. C‑2 and N‑5 (96 h): control and post-supplementation samples after supplementation ceased. Three replicates per sample

In this study, high-throughput sequencing technology was used to determine the expression levels of 6738 genes in different fermentation samples (N-1, N-2, N-3, N-4, N-5, C-1, and C-2). The distances between the control samples (C-1 and N-4, C-2 and N-5) and the ammonium sulphate supplemented samples taken at the same time were greater, indicating that our analysis focused on these two significantly different groups of samples (Figure S1). A time-based gene expression clustering analysis was performed, and the results were shown in Fig. 2. Based on the trends in gene expression, these genes were roughly categorized into 20 groups. Among these 20 expression trends, several gene clusters showed a positive correlation with the specific biosynthesis rate (*q*_p_) of nemadectin from N-1, N-2 to N-5, such as cluster 8 and cluster 19, suggesting that these genes may have been related to nemadectin biosynthesis. Moreover, some gene clusters showed a clear impact of ammonium ion supplementation on their expression trends, such as cluster 5 and cluster 13. These gene clusters were likely related to ammonium ion metabolism. After annotating and performing GO functional classification of these gene clusters, detailed studies were conducted on aspects such as cell growth, nemadectin biosynthesis, ammonium ion metabolism, and transcription factors.

#### Analysis of gene expression on nemadectin biosynthesis gene cluster

**Fig. 3:**
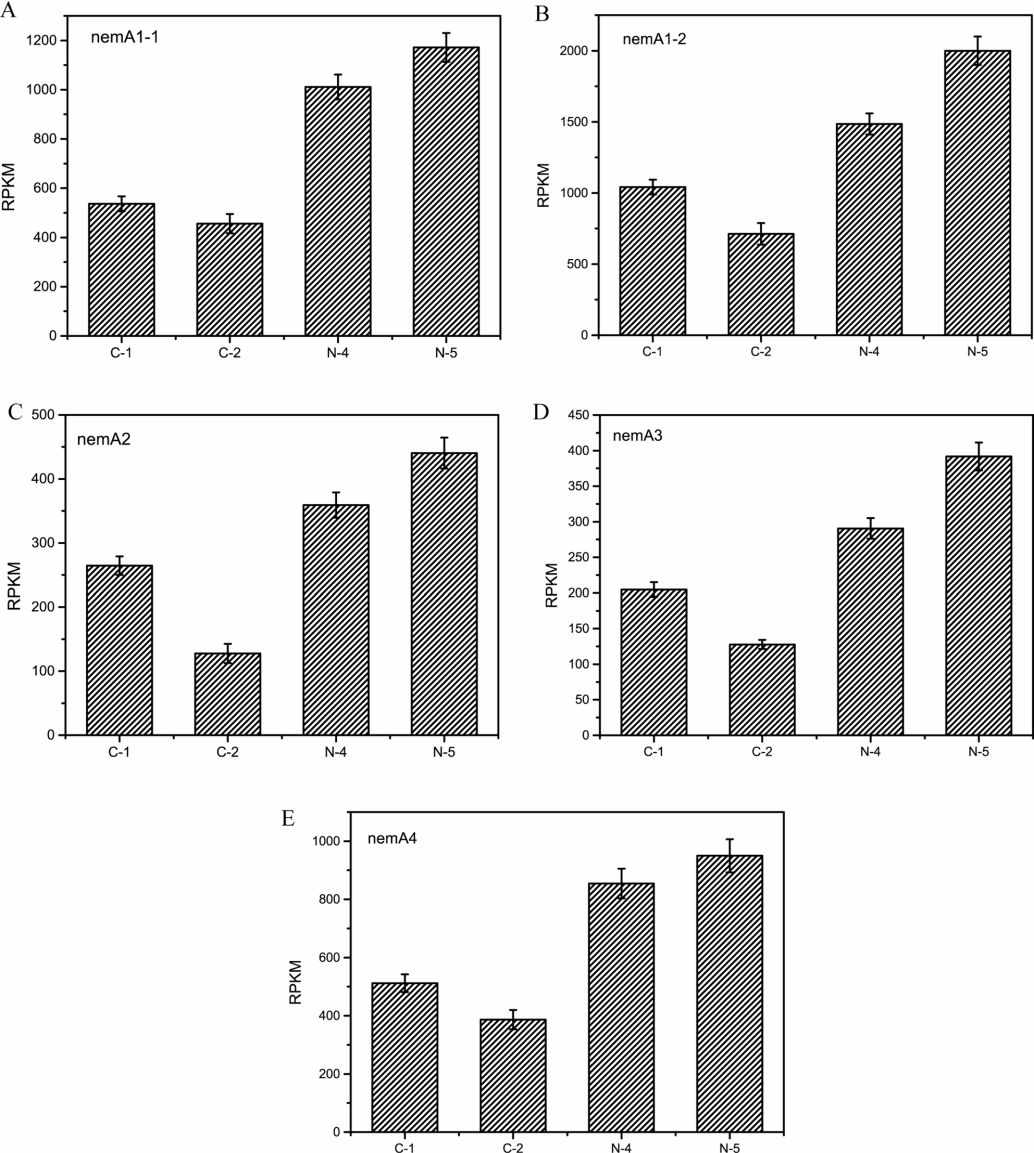
Gene expression of *nemA1-1, nemA1-2, nemA2, nemA3* and *nemA4* under different culture condition

Nemadectin is a typical type I PKS product, with genes *nemA1-1*, *nemA1-2*, *nemA2*, *nemA3* and *nemA4* encoding polyketide synthases, responsible for biosynthesizing the nemadectin scaffold from C25, C24, C23, … to C1 sequentially in the reverse direction (Figure S2). In Fig. 3, the expression trends of these five polyketide synthase-encoding genes were generally consistent. Under ammonium sulfate supplementation, the gene expression levels in samples N-4 and N-5 were significantly higher than control samples C-1 and C-2. For example, the RPKM value of *nemA1-1* reached 1011 and 1171 at N-4 and N-5, which increased 88.4% and 156.6% compared to C-1 and C-2, respectively. Additionally, in the control process, the expression levels of these genes at C-2 were lower than those at C-1, whereas under ammonium sulfate supplementation, gene expression levels at N-5 were higher than at N-4.

After the biosynthesis of the nemadectin scaffold, it requires a series of chemical modifications to form the final nemadectin. The genes involved in these modifications including *nemD*, which encoded the O-methyltransferase responsible for the modification at C5; *nemE*, which encoded the gene responsible for the formation of the furan ring between C6 and C8a; and *nemF*, which encoded the ketoreductase gene responsible for the modification at C5. Results revealed that the expression levels of these genes in samples N-4 and N-5 were significantly higher compared to the control samples C-1 and C-2 (Figure S3). Additionally, the expression levels of these modification genes in sample N-5 were also higher than in sample N-4 after ammonium sulfate supplementation. Furthermore, under ammonium sulfate supplementation, the expression of *nemR* as significantly higher in samples N-4 and N-5 (average FPKM of 790 and 731, respectively) compared with the control samples C-1 and C-2 (average FPKM of 550 and 565), representing an increase of 43.6% (N-4 vs. C-1) and 29.3% (N-5 vs. C-2), respectively (Figure S4). The *nemR* gene is located at the leftmost part of the synthesized gene cluster, and is highly homologous to the gene sequence of the avermectin *AveR* (58% homology and 70% positivity), which positively regulates nemadectin biosynthesis.

In general, secondary metabolite gene clusters in streptomyces contain one or more regulatory genes that specifically regulate the expression of genes within the cluster. These genes are known as pathway-specific regulators. Well-studied examples include *Act II-ORF4* in the *ACT* gene cluster of *Streptomyces coelicolor*, *OlmR* in the oligomycin synthesis gene cluster of *Streptomyces avermitilis* and *AveR* in the avermectin synthesis gene cluster (Fernández-Moreno et al. 1991; Guo et al. 2010). We found that under ammonium sulfate supplementation, the expression of *nemR* in samples N-4 and N-5 was significantly higher compared to the control samples C-1 and C-2 (Figure S4), indicating that ammonium sulfate supplementation benefitted the expression of *nemR*. In turn, it promoted the expression of polyketide synthase-encoding genes in the synthesis gene cluster, thereby enhancing nemadectin biosynthesis. Additionally, at the far right end of the nemadectin synthesis gene cluster, there was a gene of unknown function, *orf1*. Analysis using the Conserved Domain Database (CDD) reveals that it encoded an oxidoreductase. The expression level of this gene under ammonium sulfate supplementation (average FPKM of 200 and 187 for N‑4 and N‑5, respectively) was significantly lower than that in the control samples (average FPKM of 237 and 350 for C‑1 and C‑2) (Figure S4), a trend that contrasts with the observed increase in nemadectin titer.

#### Integrated transcriptomic and metabolomic analysis of precursors in nemadectin biosynthesis

In *Streptomyces coelicolor*, the homologous gene to *AfsKav* is TU94_RS14365 (referred to as *AfsKne* in this study) which regulates avermectin biosynthesis and influences mycelial differentiation (Rajkarnikar et al. 2006). Transcriptomic analysis revealed that during the transition from primary metabolism to secondary metabolism (N-1, N-2, and N-3 with FPKM of 94, 97, and 118, respectively), the expression level of *AfsKne* gradually increased (Figure S5). In contrast, its expression decreased during the nemadectin biosynthesis phase in the control (C‑1 and C‑2 with FPKM of 99 and 95, respectively). After ammonium sulfate supplementation, the expression level of *AfsKne* continued to rise in samples N-4 and N-5 (FPKM of 136 and 133, respectively), which is consistent with the expression trend of the polyketide synthase-encoding genes in the nemadectin synthesis gene cluster. Thus, it is likely that *AfsKne* is also regulating the synthesis of nemadectin, which is favored by an increase in its expression (Kitani et al. 2011; Uchida et al. 2011).

In *S. coelicolor*, the homologous gene to *aco* is TU94_RS00980, which can positively regulate the secondary metabolite abamectin. The expression of this gene, as shown in Figure S5, showed that its expression is higher in nemadectin biosynthesis in samples C-1 and C-2 compared to samples N-1, N-2, and N-3. Additionally, under ammonium sulfate supplementation, the expression of this gene in samples N-4 and N-5 is higher than control samples C-1 and C-2. This suggests that an Avenolide-like self-induced signaling molecule also exists in *S. coelicolor* and may have been regulating the synthesis of nemadectin. Furthermore, supplementation with ammonium sulfate facilitated the expression of the Avenolide-like signaling molecule, which in turn may be beneficial to the biosynthesis of nemadectin.

### Transcriptome-binding metabolome analysis of nemadectin synthesis precursor

The framework of nemadectin is formed through the polymerization of precursor materials by polyketide synthase. The initial building unit (C25) originates from the combination of isobutyryl-CoA and propionyl-CoA (Ikeda and Ōmura 1997). Subsequently, the PKS (polyketide synthase) system polymerizes five molecules of methylmalonyl-CoA and seven molecules of malonyl-CoA in a reverse condensation process to form nemadectin. Therefore, the synthesis of these precursors is crucial for the biosynthesis of nemadectin. This study employs a combination of transcriptomics and metabolomics to analyze the synthesis of the nemadectin precursors isobutyryl-CoA, propionyl-CoA, malonyl-CoA, and methylmalonyl-CoA.

#### Analysis of the synthesis pathway of the precursor isobutyryl coa

According to the KEGG metabolic network diagram, isobutyryl-CoA synthesis is mainly derived from valine degradation. The transcriptional expression levels of the key enzymes EC:1.4.1.23, EC:2.6.1.42 and EC:1.8.1.4 involved in valine degradation under different processes are shown in Figure S6. In samples N-4 and N-5, the expression levels of these three genes were higher than in samples C-1 and C-2, which is consistent with the transcriptional expression trends of the PKS genes in the nemadectin synthesis gene cluster. Additionally, the concentration of isobutyryl-CoA is also notably higher in samples N-4 and N-5 (0.08 and 0.23 µmol/gDCW, respectively) compared to C-1 and C-2 (both < 0.02 µmol/gDCW). Metabolomic data indicate that the intracellular concentration of valine significantly increases under ammonium supplementation, providing sufficient precursors for isobutyryl-CoA synthesis.

#### Analysis of the synthesis pathway of the precursor propionyl coa

According to the KEGG metabolic network diagram, there are four main pathways for the synthesis of propionyl-CoA: (1) propionate metabolism, (2) valine degradation, (3) isoleucine degradation and (4) alanine metabolism. Intracellular metabolite measurements revealed that the concentration of propionyl-CoA was significantly higher in samples N-4 and N-5 compared to samples C-1 and C-2, indicating that ammonium sulfate supplementation benefitted the accumulation of propionyl-CoA.

The study of the synthesis pathways identified two routes for the synthesis of propionyl-CoA from propionate metabolism. In Pathway I, the key enzyme is EC:6.2.1.1, while the key enzymes in Pathway II are EC:2.7.2.1 and EC:2.3.1.8. Analysis of the transcription levels of their coding genes revealed that the transcription levels of EC:6.2.1.1 in samples N-4 and N-5 was lower than that in samples C-1 and C-2, which differed from the transcriptional expression trend of the PKS synthase-coding genes in the nemadectin gene cluster (Zhang and Yang 2009a, b). While the transcription levels of the key enzymes EC:2.7.2.1 and EC:2.3.1.8 in Pathway II were higher in samples N-4 and N-5 compared to samples C-1 and C-2.

The key enzyme in the valine degradation pathway was EC:1.2.1.27 (Figure S7). The transcription level of this key enzyme gene in sample N-5 was significantly lower than that in sample C-2, which contrasted with the transcriptional expression trend of the PKS synthase-coding genes in the nemadectin synthesis gene cluster. Propionyl-CoA can also be synthesized through the isoleucine degradation pathway. The transcription levels of the key enzymes EC:2.6.1.42, EC:1.1.1.35 and EC:2.3.1.16 in this pathway are shown in Figure S7. In sample N-4, the transcription level of the key enzyme EC:2.3.1.16 was lower than in sample C-1, but in sample N-5, the gene expression was significantly higher than in C-2, indicating that after ammonium sulfate supplementation, the expression level of this gene increased. Additionally, the transcription levels of the key enzymes EC:2.6.1.42 and EC:1.1.1.35 were higher in samples N-4 and N-5 compared to C-1 and C-2. The intracellular concentration of isoleucine also significantly increased after ammonium sulfate supplementation, providing ample precursors for propionyl-CoA synthesis.

Finally, the transcriptional expression of the key enzymes EC:2.6.1.19, EC:4.2.1.17 and EC:1.3.1.87 in the alanine metabolism pathway showed that the transcription levels of these three key enzyme-coding genes were higher in samples N-4 and N-5 compared to samples C-1 and C-2. The concentration of alanine also significantly increased after ammonium sulfate supplementation, which indicates that ammonium sulfate supplementation enhances the alanine metabolism pathway for propionyl-CoA synthesis.

#### Analysis of the synthesis pathway of the precursor malonyl coa

Propionyl-CoA is the major precursor in the biosynthesis of nemadectin, and it is primarily synthesized through the carboxylation and condensation of acetyl-CoA by the enzyme EC:6.4.1.2 (Figure S7). Metabolomics data indicated that the concentration of acetyl-CoA was significantly higher after ammonium sulfate supplementation, providing sample precursors for the synthesis of propionyl-CoA. Analysis showed that the transcription levels of the gene encoding this key enzyme were higher in samples N-4 and N-5 compared to samples C-1 and C-2. The concentration of propionyl-CoA also significantly increased under ammonium sulfate supplementation, with concentrations of 0.0097 and 0.099 µmol/gDcw in N-4 and N-5, representing increases of 78.8% and 99.4% compared to the control samples C-1 and C-2 respectively.

#### Analysis of the synthesis pathway of the precursor methylmalonyl coa

Another important precursor in the biosynthesis of nemadectin is methylmalonyl-CoA. KEGG metabolic network diagrams indicated that the synthesis primarily occurs from three pathways: (1) carboxylation of isobutyryl-CoA, (2) carboxylation of propionyl-CoA, (3) rearrangement of succinyl-CoA (Figure S8). Intracellular metabolite measurements revealed that the concentration of the precursor methyl-malonyl-CoA significantly increased under ammonium supplementation with concentrations of 0.013 and 0.085 µmol/gDcw in samples N-4 and N-5 respectively, representing increases of 29.6% and 288.6% compared to control samples C-1 and C-2. This preliminary result suggests that ammonium sulfate supplementation is beneficial for the synthesis of methyl-malonyl-CoA.

Next, we investigated the metabolic pathways involved in the synthesis of methyl-malonyl-CoA. As shown in Figure S9, the key enzyme in the carboxylation of isobutyryl-CoA to synthesize methyl-malonyl-CoA is EC:1.2.1.3. The transcription levels of the gene encoding this enzyme were lower in sample N-5 compared to sample C-2.

Subsequently, analysis of the key enzyme EC:6.4.1.3 in the propionyl-CoA carboxylation pathway showed that the transcription levels of the gene encoding this enzyme were higher in samples N-4 and N-5 compared to C-1 and C-2. Additionally, the concentration of propionyl-CoA under ammonium supplementation conditions was higher than control. Furthermore, in the succinyl-CoA rearrangement pathway, the key enzymes were EC:5.4.99.2 and EC:5.4.99.1 (Figure S9). The transcription levels of the genes encoding these two enzymes were lower in samples N-4 and N-5 compared to C-1 and C-2.

#### Analysis of the synthesis pathway of the precursor acetyl coa

**Fig. 4:**
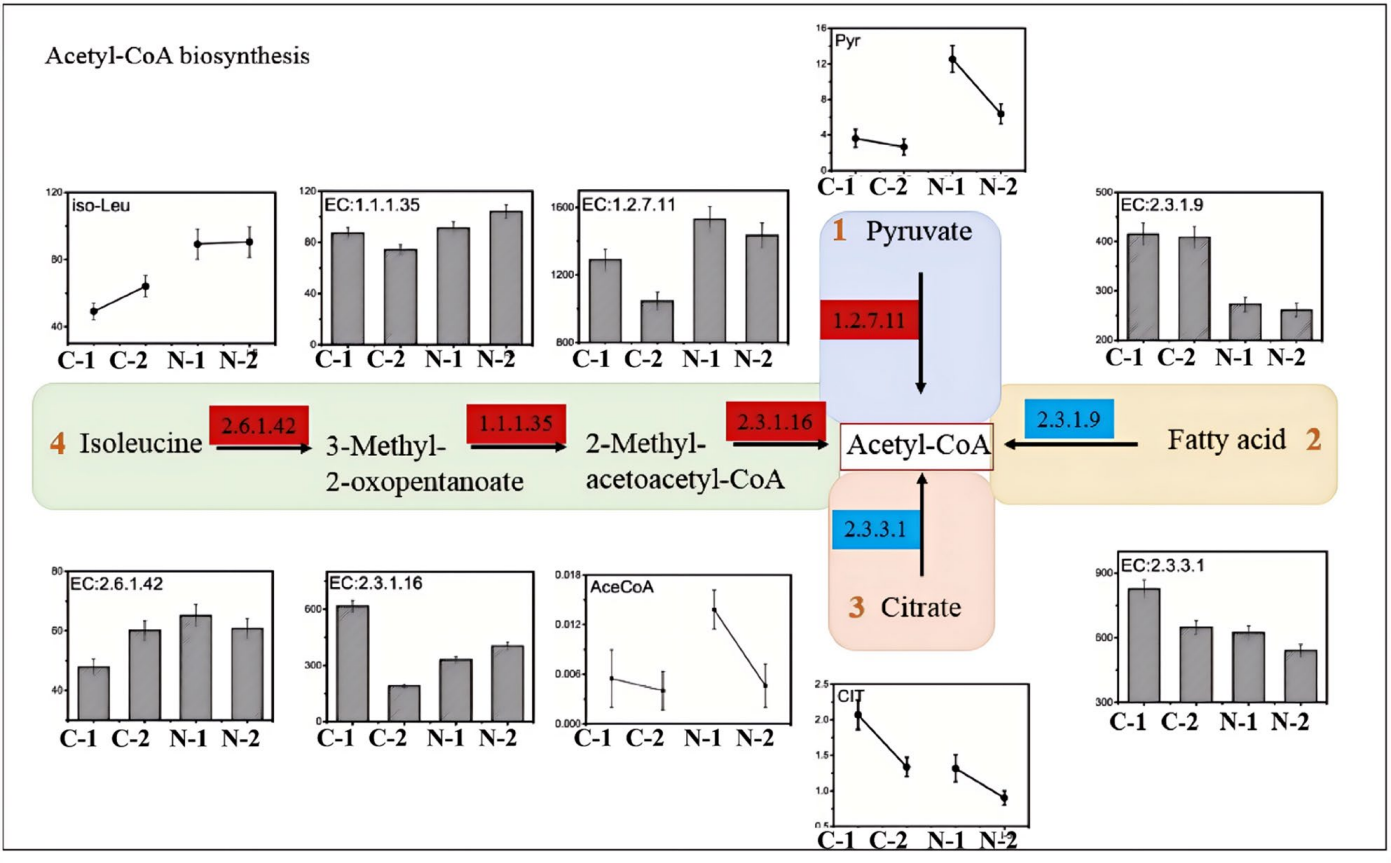
The synthetic route of Acetyl-CoA in *S. cyaneogriseus* (red /blue line indicated the pathway was enhanced/decreased after supplementation ammonium sulfate. The unit of vertical axis is µmol/gDcw for intracellular metabolites)

Acetyl-CoA is a central metabolic hub whose carbon skeleton can be derived from multiple catabolic pathways. In the metabolic context of *S. cyaneogriseus*, major contributing routes likely include the decarboxylation of pyruvate and the β-oxidation of fatty acids. Additionally, the degradation of certain amino acids (e.g., isoleucine) and other substrates may also contribute to the acetyl-CoA pool (Fig. 4). Intracellular metabolite measurements revealed that the concentration of acetyl-CoA in samples N-4 and N-5 was significantly higher than in samples C-1 and C-2, indicating that ammonium sulfate supplementation is beneficial for acetyl-CoA synthesis.

As shown in Fig. 4, pyruvate can be metabolically converted into acetyl-CoA. We analyzed the transcriptional expression levels of the key enzyme EC:1.2.7.11 in this pathway and found that the transcription levels of this gene in samples N-4 and N-5 were higher than in C-1 and C-2. Metabolomic analysis also indicated that pyruvate concentrations in samples N-4 and N-5 were significantly higher than in C-1 and C-2, providing more precursors for acetyl-CoA synthesis through this metabolic pathway.

In contrast, the key enzyme in the fatty acid degradation pathway is EC:2.3.1.9, and the transcription levels of the gene encoding this enzyme were lower in samples N-4 and N-5 compared to C-1 and C-2. For the reverse TCA cycle pathway, the key enzyme EC:2.3.3.1 had lower expression levels in samples N-4 and N-5 compared to C-1 and C-2. Additionally, the intracellular citrate concentration was significantly lower under ammonium supplementation.

Finally, we analyzed the transcriptional expression of key enzymes EC:2.6.1.42, EC:1.1.1.35 and EC:2.3.1.16 in the isoleucine degradation pathway. The transcription level of the EC:2.3.1.16 gene was lower in sample N-4 compared to C-1, but higher in sample N-5 compared to C-2, indicating that the expression was elevated following ammonium sulfate supplementation. The other two key enzyme genes had higher transcription levels in samples N-4 and N-5 than in C-1 and C-2. Additionally, the concentration of isoleucine was significantly higher under ammonium supplementation.

### Transcriptome-bound metabolome analysis of intracellular amino acid biosynthesis

The biosynthesis metabolism of amino acids is closely related to the growth of microbial cells and the biosynthesis of nemadectin precursor coenzyme A-type substances. To investigate the biosynthesis of intracellular amino acids under the ammonium sulfate supplementation, we analyzed the changes in key amino acids in samples N-4 and N-5 with ammonium sulfate supplementation compared to control samples C-1 and C-2 from transcriptomic and metabolomic perspectives. As shown in Figure S10, with the exception of Asp, Gln, Met, Pro and Ser, all the other amino acids showed a significant increase following the addition of ammonium sulfate. The increase in amino acid concentrations can provide more nutrients for the growth of the cells, which may be one of the reasons for the increase in biomass under the ammonium sulfate supplementation process. On the other hand, amino acids serve as the main precursors of coenzyme A-type substances. There was a noticeable increase in the synthesis precursors of acetyl-CoA and propionyl-CoA, including Ile (for the synthesis of isobutyryl-CoA), Val (for the synthesis of acetyl-CoA) and Ala (for the synthesis of propionyl-CoA). This indicates that the addition of ammonium sulfate is beneficial for the accumulation of precursor amino acids in the cells of *Streptomyces lavendulum*, providing more precursors for the biosynthesis of coenzyme A-type substances, thereby promoting nemadectin biosynthesis.

This study also analyzed the changes in the expression trends of key genes in different amino acid biosynthesis pathways using transcriptomic data. As shown in Figure S11, with the exception of a few amino acids such as Gln, Pro and Ser, the expression levels of other amino acids in samples N-4 and N-5 under ammonium sulfate supplementation were higher than in samples C-1 and C-2. Among them, the precursors for coenzyme A-type substances such as Ile, Val and Ala, showed increases following ammonium sulfate supplementation (Amon et al. 2008; Liao et al. 2015).

### Transcriptome-combined metabolome analysis of central carbon metabolism pathway under ammonium supplementation process

**Fig. 5:**
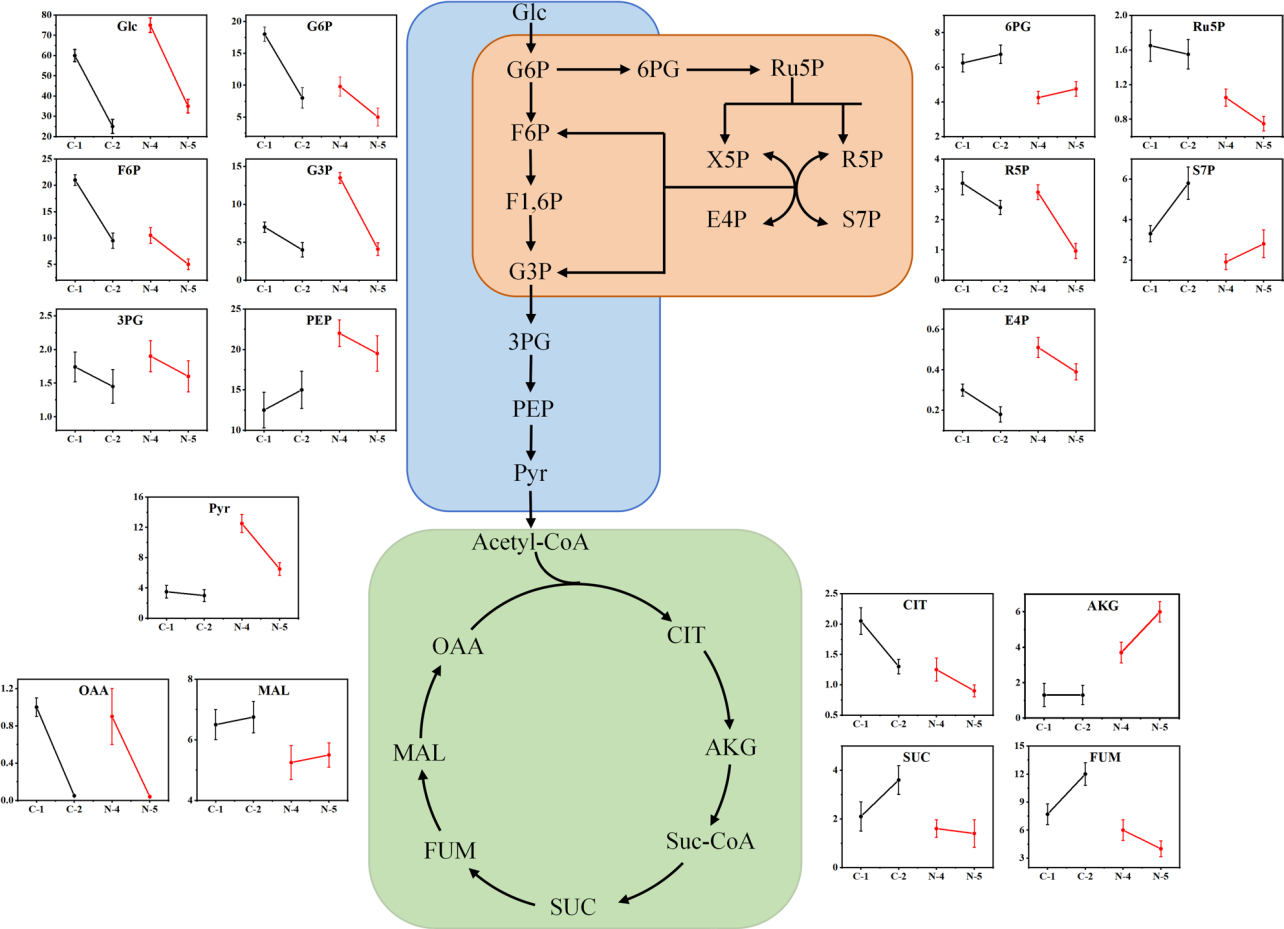
Profiles of metabolite concentration of organic acid and sugar phosphates under different culture condition. The unit of vertical axis is µmol/gDcw for intracellular metabolites. C-1 and N-4 (60 h): control and supplemented samples during ammonium supplementation. C-2 and N-5 (96 h): control and post-supplementation samples after supplementation ceased. Three replicates per sample

The Embden–Meyerhof–Parnas (EMP) pathway provides a small amount of ATP and essential growth precursors for cell growth. This pathway involves three key enzymes: hexokinase (EC:2.7.1.63), phosphofructokinase (EC:2.7.1.11) and pyruvate kinase (EC:2.7.1.40) (Tiffert et al. 2011). As shown in Figure S12, the expression levels of the genes encoding these three key enzymes were higher in samples N-4 and N-5 than in the control samples C-1 and C-2 under ammonium sulfate supplementation, indicating that ammonium sulfate supplementation may increase the metabolic flux through the EMP pathway. Additionally, from the metabolite pool data (Fig. 5), it was observed that the intracellular Glc concentration under ammonium sulfate supplementation was higher compared to the control, suggesting an increased glucose uptake by the cells. Furthermore, the concentrations of upstream metabolites G6P and F6P in the EMP pathway were lower in samples N-4 and N-5 under ammonium sulfate than in C-1 and C-2. This may be due to the increased expression of key upstream enzymes, hexokinase and phosphofructokinase, which accelerated the conversion of upstream metabolites to downstream products (Yao et al. 2014). It also shows that the concentrations of downstream metabolites 3PG, PEP, and Pyr in the EMP pathway were higher in samples N-4 and N-5 compared to C-1 and C-2. Specifically, Pyr concentrations in N-4 and N-5 reached 12.54 and 6.38 µmol/gDcw, representing increases of 245.3% and 141.7% over the control samples C-1 and C-2, respectively.

The pentose phosphate (PP) pathway is a source of nucleotide synthesis precursors and NADPH and is closely related to cell biomass synthesis. The first step of this pathway is the rate-limiting step. As shown in Figure S12, the expression level of the gene encoding glucose-6-phosphate dehydrogenase (EC:1.1.1.363), which catalyzes the first step from G6P to 6GP, was significantly higher in sample N-4 compared to C-1. Similarly, the gene expression in N-5 was slightly higher than in C-2. Furthermore, except for the genes encoding EC:1.1.1.44 and EC:5.3.1.1, the expression levels of other genes in the pathway were higher in samples N-4 and N-5 under ammonium sulfate supplementation compared to controls C-1 and C-2. Metabolite concentrations indicate that most metabolites in the pathway were lower in samples N-4 and N-5 compared to C-1 and C-2 with ammonium sulfate supplementation. While this is challenging to explain based solely on the PP pathway, combining with EMP pathway analysis reveals that G3P is higher in samples N-4 and N-5 compared to C-1 and C-2 (opposite to the trend observed for upstream metabolites in the EMP pathway).

It can be observed that the expression levels of almost all genes in the TCA cycle were lower in samples N-4 and N-5 compared to the control samples C-1 and C-2. Metabolomic data also showed that the concentrations of major TCA cycle products (except AKG) were lower in N-4 and N-5 compared to C-1 and C-2 (Fig. 5). This indicates that ammonium sulfate supplementation may lead to reduction in TCA cycle flux, resulting in decreased flow of Pyr from the EMP pathway to the TCA cycle.

### Potential regulatory links between nitrogen metabolism and nemadectin biosynthesis

In addition to pathway-specific regulation, the synthesis of secondary metabolites in *Streptomyces* may also be modulated by global transcriptional regulators (Zhu et al. 2017). In this study, under conditions of ammonium sulfate supplementation, the expression of the pathway-specific positive regulator for nemadectin, *nemR*, showed an upregulation trend. Concurrently, we focused on GlnR, a key global transcriptional regulator of nitrogen metabolism. GlnR is responsible for coordinating nitrogen uptake and assimilation, facilitating the conversion of various extracellular nitrogen sources into primary intracellular nitrogen donors such as glutamine and glutamate, thereby playing a vital role in cellular metabolism (Tiffert et al. 2011).

As shown in Fig. S13, the expression of *glnR* is tightly regulated by the ammonium concentration in the medium: its expression is repressed under ammonium-sufficient conditions (e.g., post-supplementation samples N-4, N-5), while it is significantly induced under nitrogen-limited conditions following ammonium depletion (e.g., samples N-2, C-1, C-2). This observation aligns with existing literature. Furthermore, GlnR orchestrates the expression of downstream genes involved in nitrogen assimilation (Fig. S13). Under nitrogen limitation, it activates genes responsible for nitrogen acquisition and assimilation, such as the ammonium transporter gene (*amt*), glutamine synthetase genes (*glnA*, *glnII*), as well as those for nitrite reductase (*nirB*) and urease. Correspondingly, under ammonium-sufficient conditions following supplementation, the expression of these GlnR-dependent genes (e.g., *amt*, *glnA*, *glnII*, *nirB*) is strongly repressed. In contrast, the expression of the glutamate dehydrogenase gene (*gdhA*) is markedly upregulated under these conditions (Fig. S14).

Metabolite data corroborate these findings (Fig. S15). Under ammonium-supplemented conditions (N-4, N-5), the intracellular glutamate concentration was significantly higher than in the control, while glutamine levels were lower. This indicates that under the ammonium-sufficient conditions provided by supplementation, the cellular nitrogen assimilation pathway shifts from the ATP-dependent GS/GOGAT pathway (positively regulated by GlnR) to the more economical GDH pathway, leading to direct glutamate synthesis. This metabolic flux redirection may provide a more ample supply of precursor amino acids and a more favorable energy charge for nemadectin biosynthesis.

### Speculated mechanism of ammonium induced nemadectin biosynthesis

**Fig. 6:**
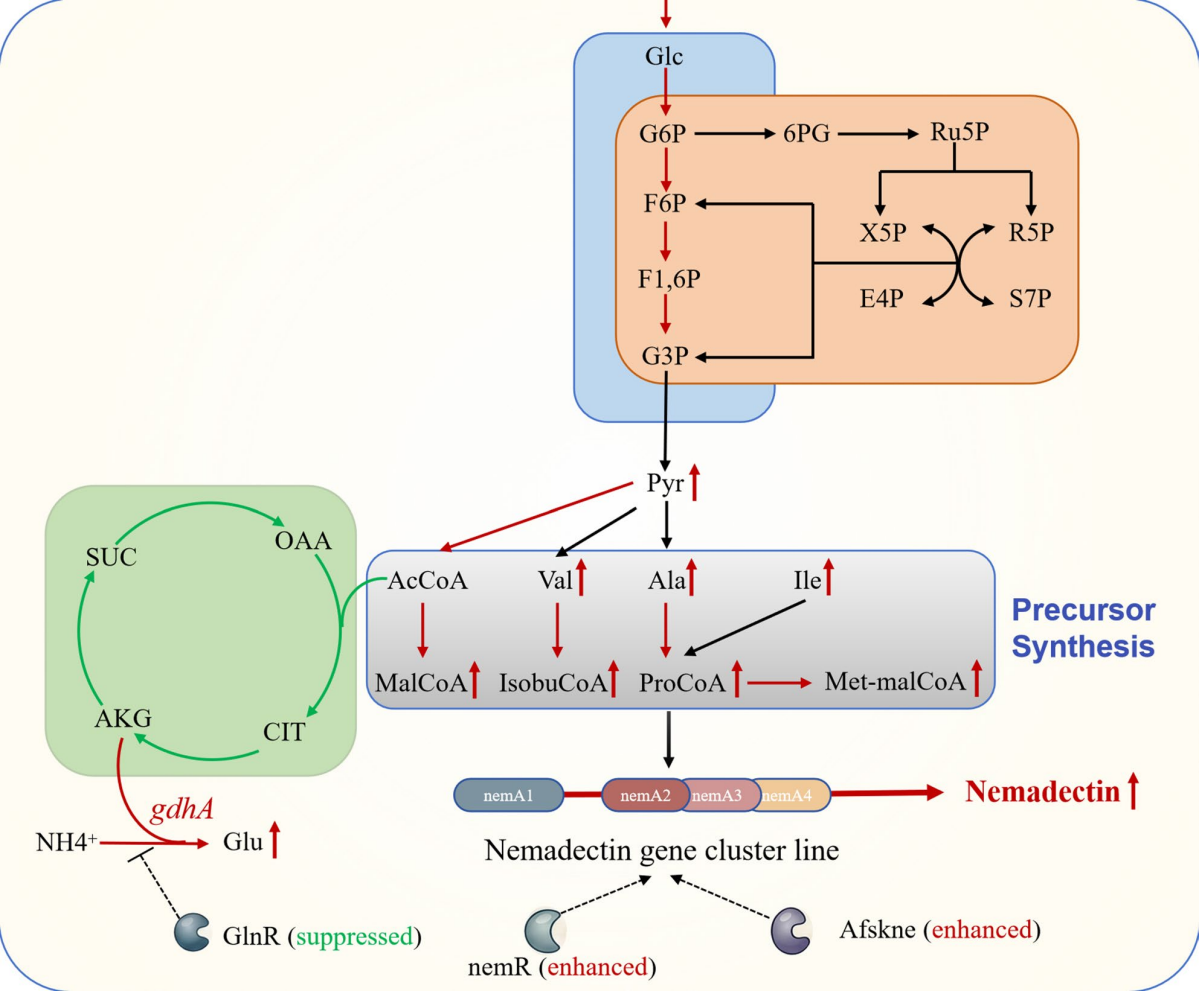
Mechanism of high nemadectin accumulation under ammonium sulfate supplementation. In the diagram, a red arrow pointing to a metabolite indicates an increase in its intracellular pool. A red arrow along a metabolic pathway denotes upregulation of the corresponding genes. A green arrow indicates downregulation of gene expression. Dashed arrows represent putative positive regulation by a transcription factor, while dashed arrows with a perpendicular bar represent putative negative regulation. The terms “suppressed” and “enhanced” in parentheses following a transcription factor indicate decreased or increased expression of its encoding gene, respectively

Integrated transcriptomic and metabolomic analyses revealed the following mechanism for ammonium-induced nemadectin biosynthesis (Fig. 6): (1) Intracellular concentrations of the nemadectin precursors isobutyryl-CoA, propionyl-CoA, succinyl-CoA, and methylmalonyl-CoA significantly increased, indicating enhanced synthesis of these compounds. (2) The concentrations and synthesis pathways of most amino acids, particularly isoleucine (a precursor of acetyl-CoA), valine (a precursor of isobutyryl-CoA), and alanine (a precursor of propionyl-CoA), were strengthened under ammonium supplementation. This provided ample precursors for CoA derivatives while supporting microbial growth through increased biomass. (3) Analysis of central carbon metabolism indicated increased flux through the EMP and PP pathways under ammonium supplementation, benefiting microbial growth. Concurrently, TCA cycle flux decreased, redirecting pyruvate towards nemadectin precursor synthesis. (4) Ammonium sulfate supplementation triggered the formation of glutamic acid from ammonium ions and α-ketoglutarate, catalyzed by glutamic acid dehydrogenase. The pathway converting glutamic acid to pyruvate was enhanced, with subsequent pyruvate metabolism producing acetyl-CoA, valine, isoleucine, and alanine. This coordinated metabolic shift under ammonium supplementation generated sufficient precursors for nemadectin biosynthesis.

## Conclusions

This study investigated the high-yield mechanism of nemadectin production under ammonium sulfate supplementation by analyzing physiological phenotype data, metabolomics, and transcriptomics. Transcriptomic data revealed a significant increase in the expression of genes within the nemadectin biosynthesis gene cluster, including genes encoding PKS, nemadectin backbone modification genes, and pathway-specific transcription factors. This indicates that ammonium sulfate supplementation enhances nemadectin biosynthesis via PKS by boosting precursor acetyl-CoA biosynthesis. Furthermore, the high expression of genes related to Avenolide-like signaling molecules and global transcription factor *Afskne* suggests that ammonium supplementation also enhances the expression of these regulatory elements, thereby positively upregulating nemadectin biosynthesis.

## Electronic Supplementary Material

Below is the link to the electronic supplementary material.

Supplementary Material 1

## Data Availability

The datasets used and/or analysed during the current study are available from the corresponding author on reasonable request.

## Abbreviations

EMP: Embden–Meyerhof–Parnas
TCA: Tricarboxylic acid cycle
PBS: Phosphate buffered solution
PKS: Polyketide synthase
KEGG: Kyoto encyclopedia of genes and genomes
G6P: Glucose-6-phosphate
6GP: 6-Glucose-phosphate
F6P: Fructose-6-phosphate
3PG: 3-Phosphoglycerate
G3P: Glyceraldehyde-3-phosphate
PEP: Phosphoenolpyruvate
PP: Protein phosphatase
AKG: α-Ketoglutaric acid
Dcw: Dry cell weight
Pyr: Pyruvate
Asp: Aspartic acid
Gln: Glutamine
Met: Methionine
Pro: Proline
Ser: Serine
