# Supplementary material for: Mechanistic insights into ammonium-driven metabolic regulation for enhanced nemadectin biosynthesis in Streptomyces cyaneogriseus
Source: Bioresour Bioprocess. 2026 Jan 30;13(1):20. doi: 10.1186/s40643-026-01015-6 (PMC12858678; doi:10.1186/s40643-026-01015-6)
Supplement: Supplementary file 1 — Supplementary Material 1 [file 40643_2026_1015_MOESM1_ESM.docx]

**Supporting information**

**Mechanistic Insights into Ammonium-Driven Metabolic Regulation for Enhanced Nemadectin Biosynthesis in *Streptomyces cyaneogriseus***

Zishu Zhang^1#, b^, Junxiong Yu^1#,^ ^a, *^, Xiaoqing Song^a^, Qingfeng Gu^a^, Yun Zhang^a^, Jiayun Xue^a^, Ali Moshin^a^, Yonghong Wang^a^, Zejian Wang^a,^ *

1. State Key Laboratory of Bioreactor Engineering, East China University of Science and Technology, 130 Meilong Rd., Shanghai 200237, China
2. State Key Laboratory of Microbial Metabolism, School of Life Sciences and Biotechnology, Joint International Research Laboratory of Metabolic and Developmental Sciences, Shanghai Jiao Tong University, 800 Dongchuan Rd, Shanghai 200240, China

**Dr. Junxiong Yu**

Address: East China University of Science and Technology, 130 Meilong Rd., Shanghai 200237, P. R. China. Tel: +86 21 64253011; Fax: +86 21 64252252.

E-mail: yjxecuster@163.com **(J. Yu)**

**Professor Dr. ZeJian Wang**

Address: East China University of Science and Technology, 130 Meilong Rd., Shanghai 200237, P. R. China. Tel: +86 21 64253011; Fax: +86 21 64253702.

E-mail: wangzejian@ecust.edu.cn **(Z. Wang)**

**Supporting Figure**


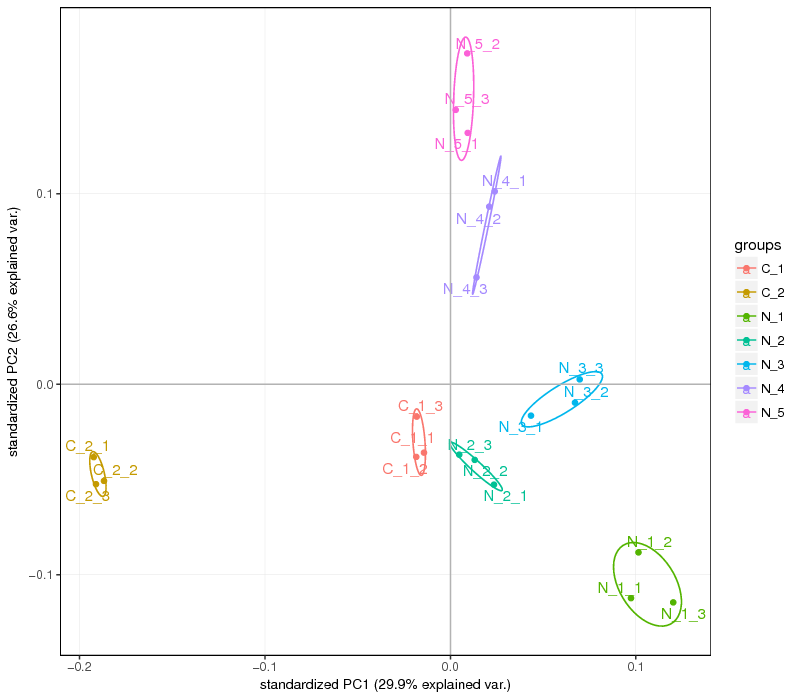


**Fig.S1** PCA analysis of the RNA-seq samples

**Fig.S2** The regulation gene cluster of nemadectin biosynthesis in *S. cyaneogriseus*


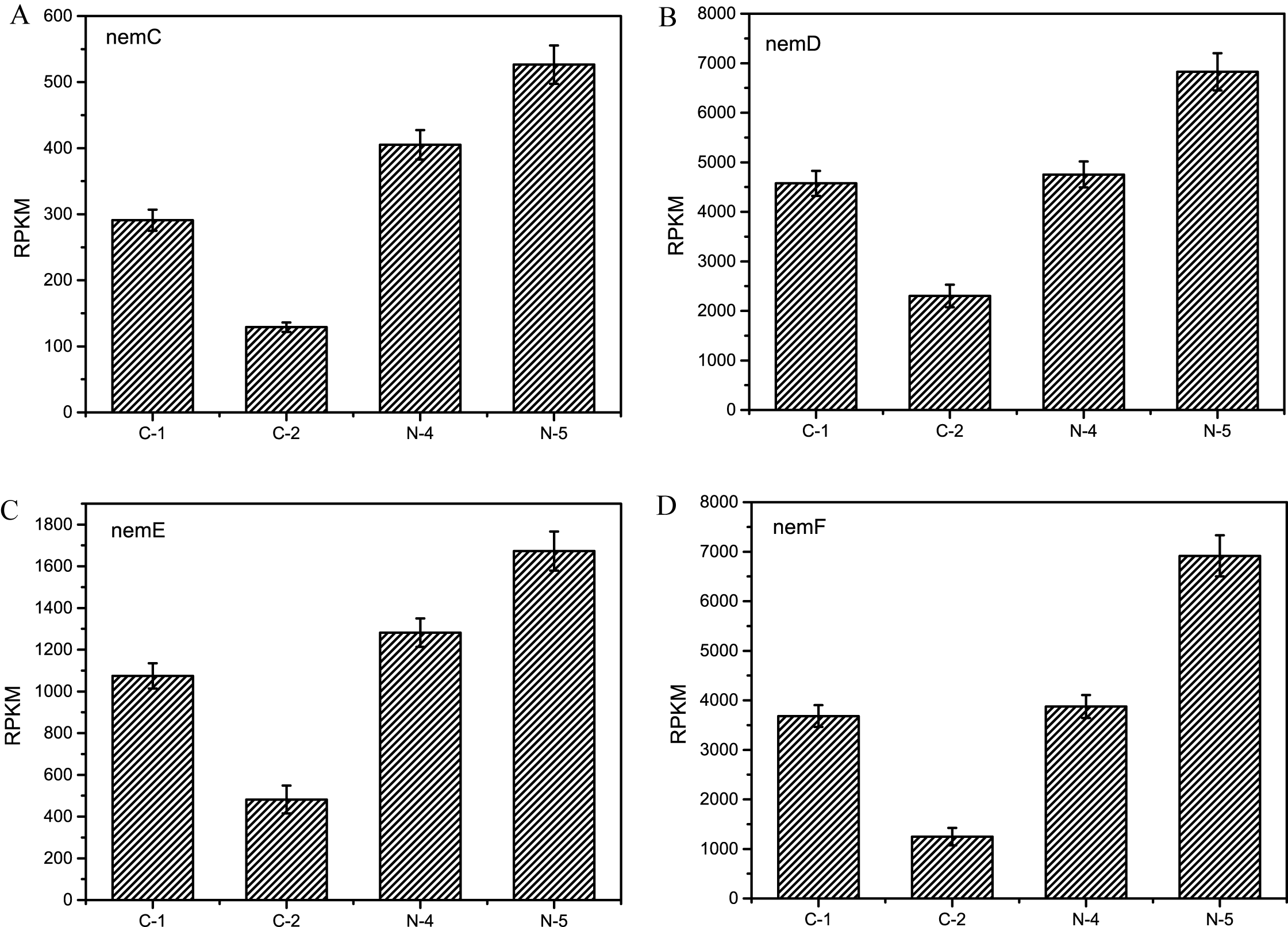


**Fig.S3**  Gene expression of the nemadectin modifying gene under different culture condition


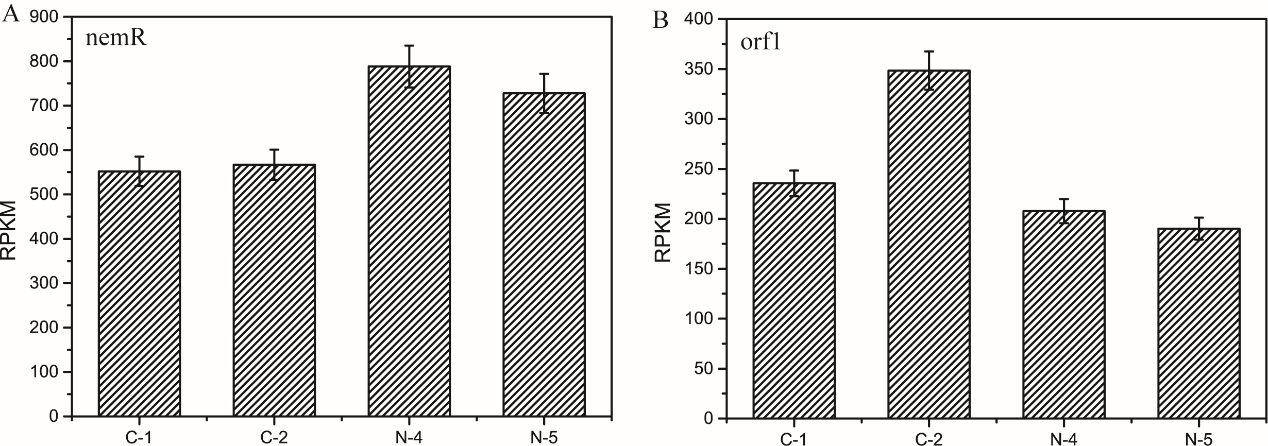


**Fig.S4** Gene expression of *nemR* and *orf1* under different culture condition


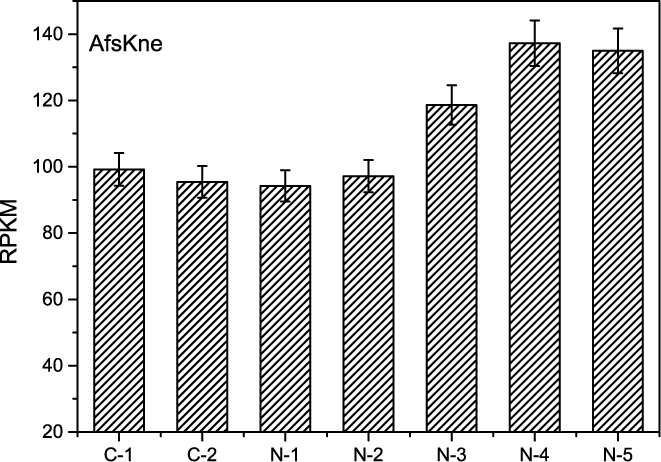


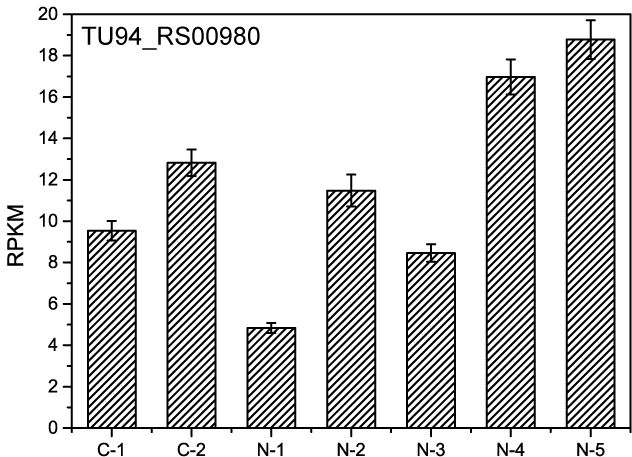


**Fig.S5** Gene expression of *AfsKne* and *aco* homologous gene under different culture condition


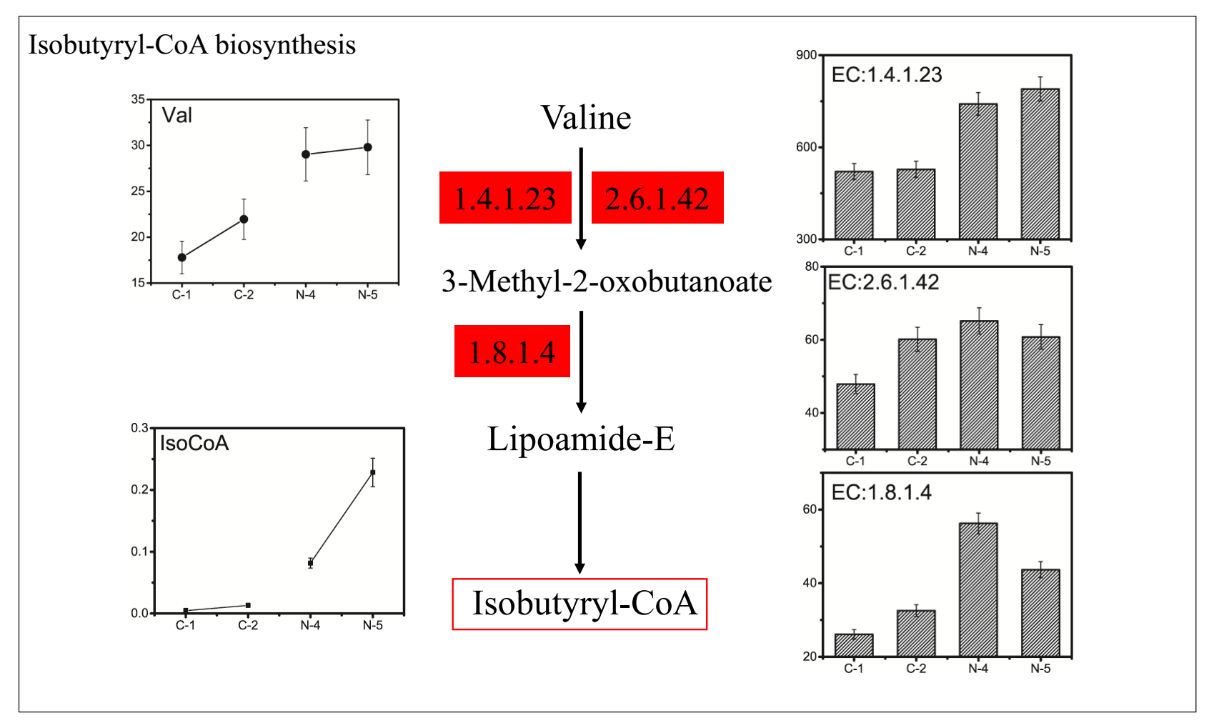


**Fig.S6** The synthetic route of Isobutyryl-CoA in *S. cyaneogriseus* (red line indicated the pathway was enhanced after supplementation ammonium sulfate, the unit of vertical axis is μmol/gDcw for intracellular metabolites )


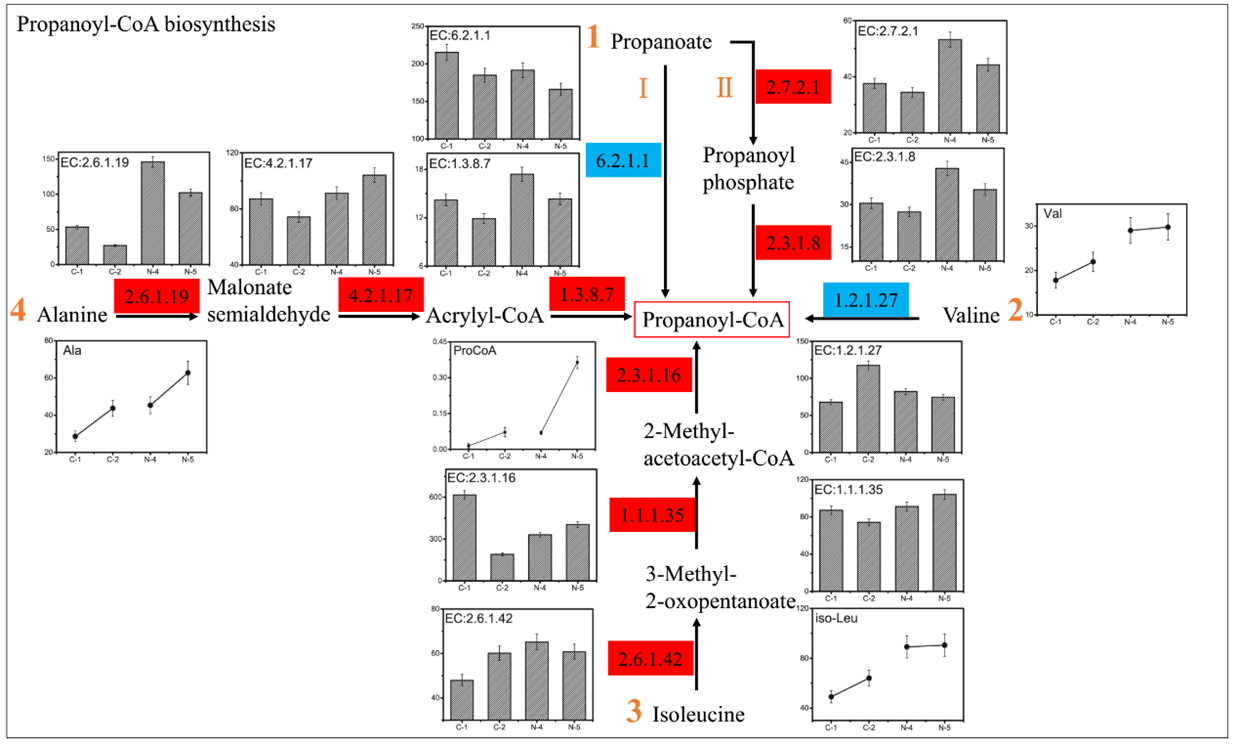


**Fig.S7** The synthetic route of propanoyl-CoA in *S. cyaneogriseus* (red /blue line indicated the pathway was enhanced/decreased after supplementation ammonium sulfate, the unit of vertical axis is μmol/gDcw for intracellular metabolites)


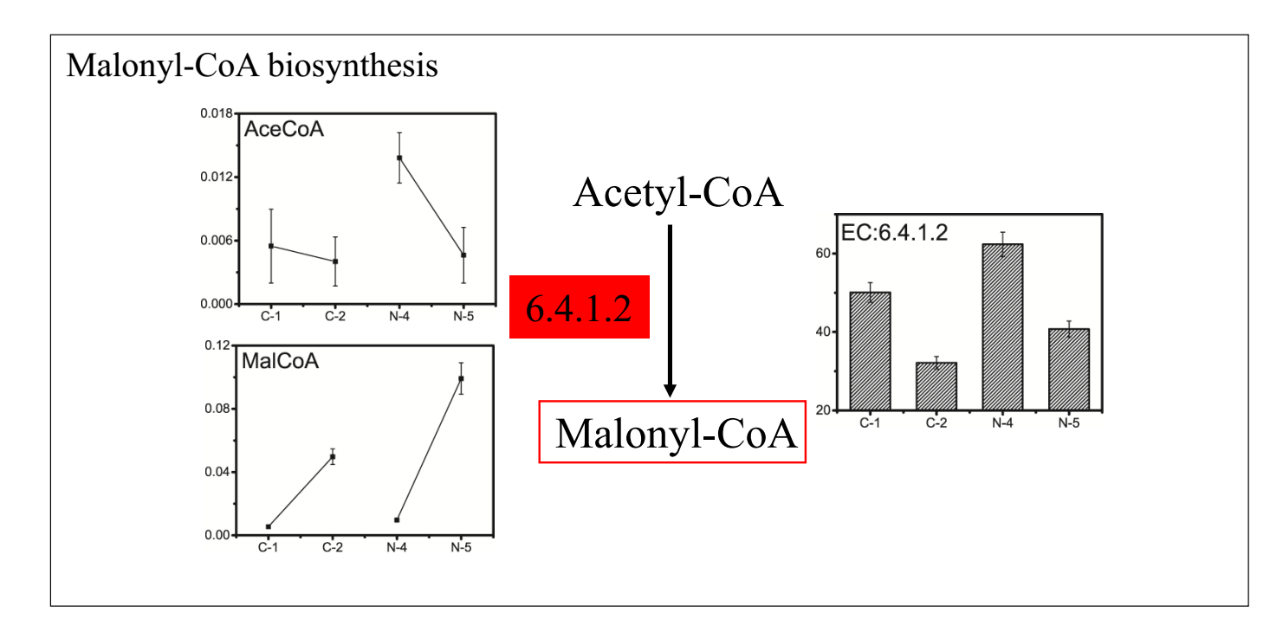


**Fig.S8** The synthetic route of Malonyl-CoA in *S. cyaneogriseus* (red line indicated the pathway was enhanced after supplementation ammonium sulfate, the unit of vertical axis is μmol/gDcw for intracellular metabolites)


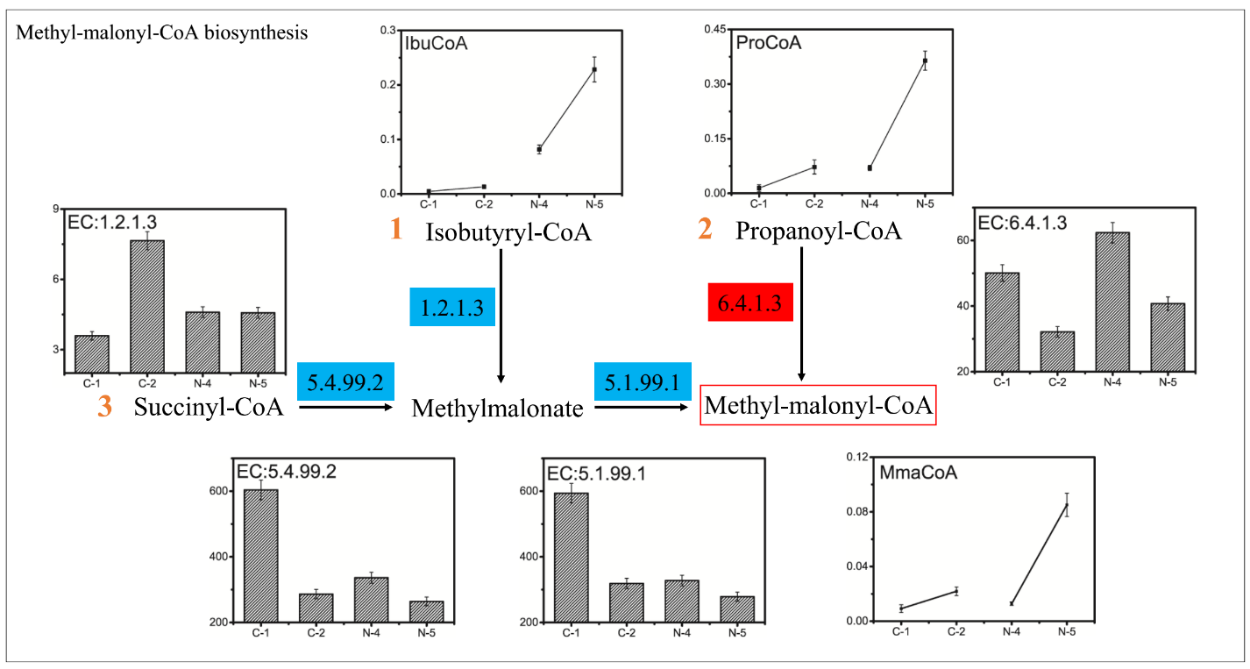


**Fig.S9** The synthetic route of Methyl-malonyl-CoA in *S. cyaneogriseus* (red /blue line indicated the pathway was enhanced/decreased after supplementation ammonium sulfate. the unit of vertical axis is μmol/gDcw for intracellular metabolites)


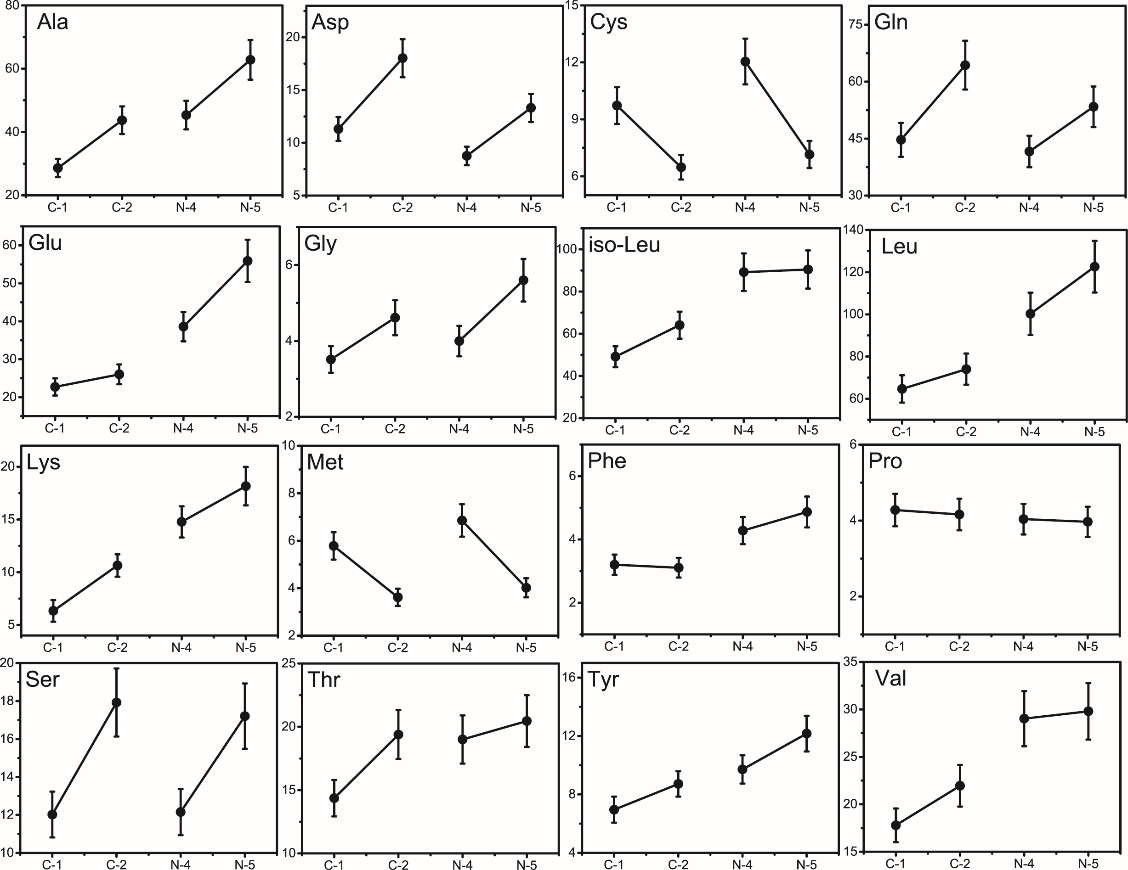


**Fig.S10** Changes in intracellular amino acids concentration under different fermentation condition. The sample number is on the horizontal axis and the amino acids concentration is on the ordinate axis (Unit: μmol/gDcw).


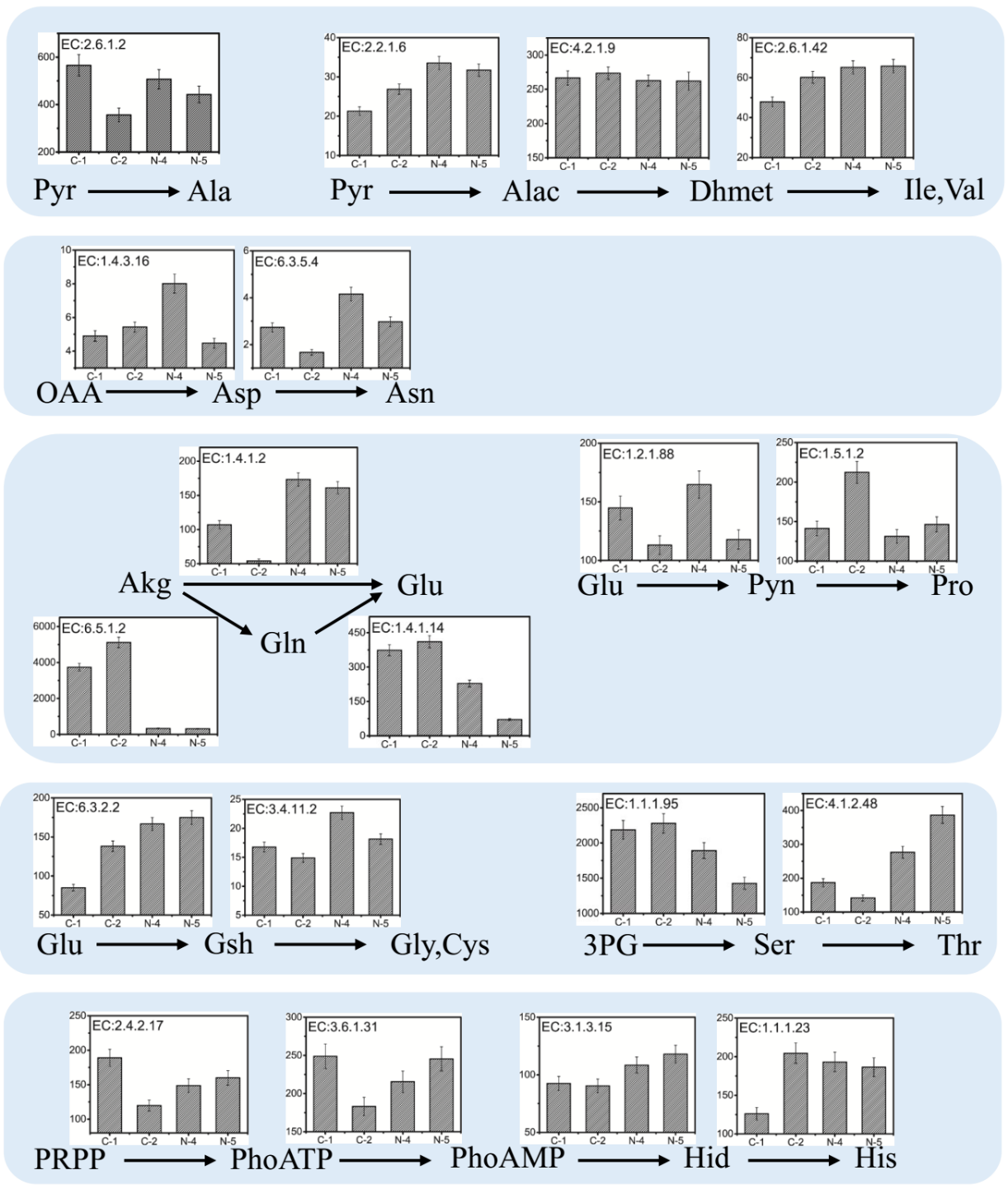


**Fig.S11** The tendencies of gene expressin on synthesis pathway of amino acids in different culture condition.


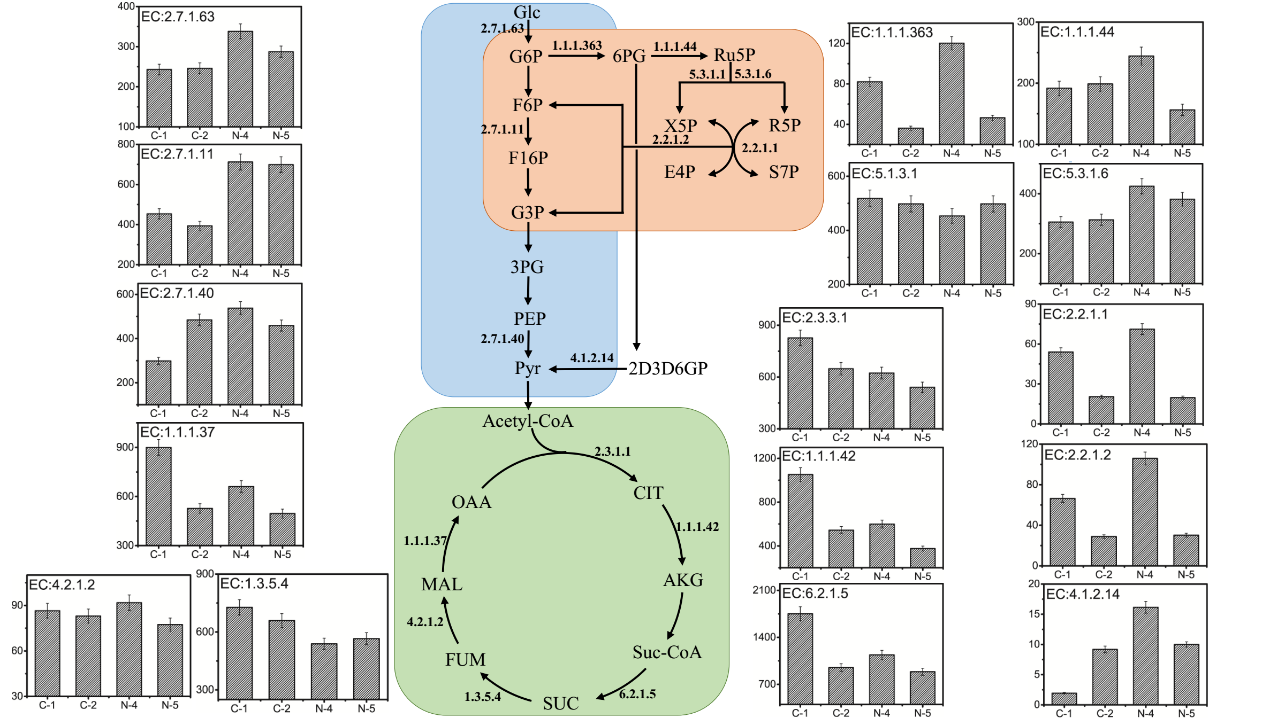


**Fig.S12** Changes in tendencies of genes expression and relative flux on central carbon metabolism under different culture condition.


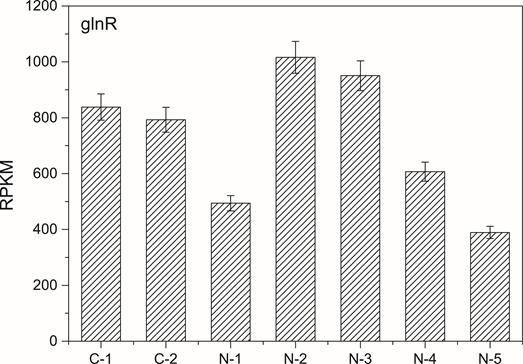


**Fig.S13** Gene expression of *glnR* under different culture condition


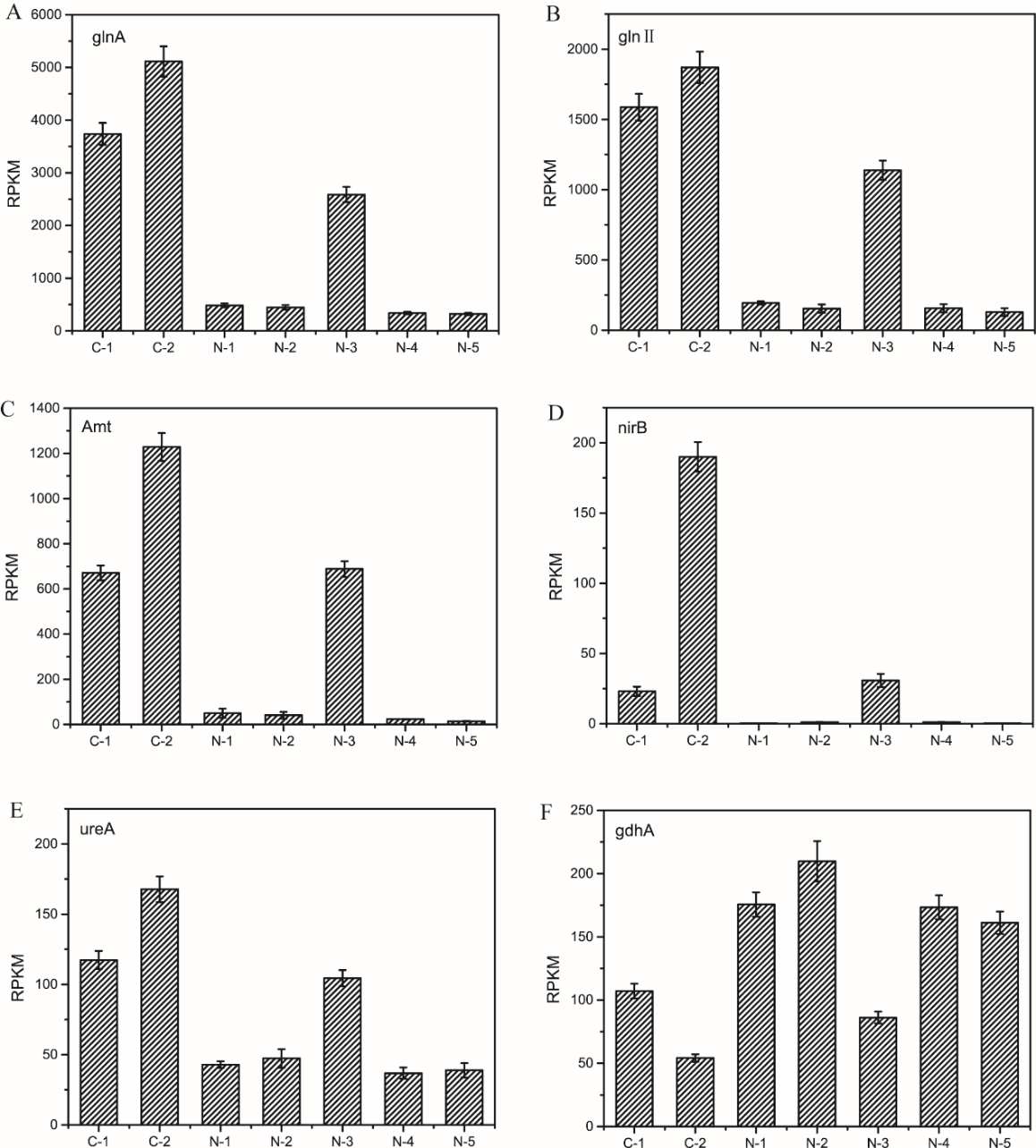


**Fig.S14** The expression of genes regulated by *glnR* under different culture condition


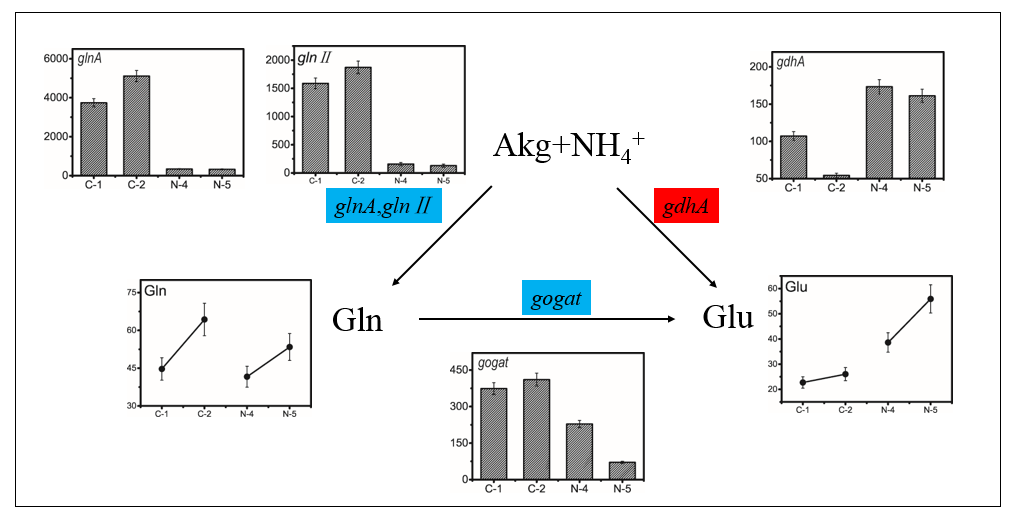


**Fig.S15** Ammonium ion metabolism pathway after supplementation ammonium sulfite. (red/blue line indicated the pathway was enhanced/decreased after supplementation ammonium sulfate, the unit of vertical axis is μmol/gDcw for intracellular metabolites)
